# Supplementary material for: Regression with Highly Correlated Predictors: Variable Omission Is Not the Solution
Source: Int J Environ Res Public Health. 2021 Apr 17;18(8):4259. doi: 10.3390/ijerph18084259 (PMC8073086; doi:10.3390/ijerph18084259)
Supplement: Supplementary file 1 [file ijerph-18-04259-s001.zip › Paper___Climate_Change_SuppM1_V10.pdf]

Supplementary Material 1

Regression with highly correlated predictors: variable omission is not the solution

Section S1: Additional information regarding the blood analysis data

Table S1. Full subset of the data from the study of Ratzinger et al. (2014) used for the blood cell example in the paper. White blood cells consist of the five subtypes neutrophils eosinophils, basophils, lymphocytes and monocytes and hence, their sum equals the white blood cell count.

Observation	Neutrophils (G/L)	Eosinophils (G/L)	Basophils (G/L)	Lymphocytes (G/L)	Monocytes (G/L)	White blood cell count (G/L)	C-reactive protein (mg/dL)
1	11.5	0.0	0.1	0.6	1.1	13.3	15.99
2	13.9	0.0	0.0	3.0	3.3	20.2	13.27
3	13.0	0.2	0.0	0.2	1.1	14.5	14.99
4	11.0	0.1	0.0	0.6	0.8	12.5	9.93
5	10.1	0.0	0.0	0.6	0.8	11.5	16.70
6	4.2	0.0	0.0	1.0	0.9	6.1	7.74
7	11.9	0.2	0.0	1.4	1.0	14.5	10.13
8	4.5	0.2	0.0	0.7	0.6	6.0	4.86
9	3.5	0.0	0.0	0.5	0.0	4.0	15.02
10	6.4	0.2	0.0	1.2	0.6	8.4	3.76
11	0.0	0.0	0.0	0.4	0.0	0.4	12.53
12	3.5	0.0	0.0	0.3	0.4	4.2	15.72
13	0.0	0.0	0.1	1.0	0.5	1.6	1.39
14	10.5	0.0	0.0	1.2	1.3	13.0	7.66
15	0.4	0.1	0.0	0.3	0.1	0.9	7.09
16	7.5	0.0	0.0	1.9	0.6	10.0	10.34
17	4.9	0.0	0.0	0.3	0.3	5.5	16.58
18	6.1	0.4	0.1	1.4	0.8	8.8	6.09
19	9.5	0.3	0.0	1.4	0.9	12.1	5.01
20	1.3	0.0	0.0	0.3	0.2	1.8	8.28

Section S2: Additional information regarding the climate change example

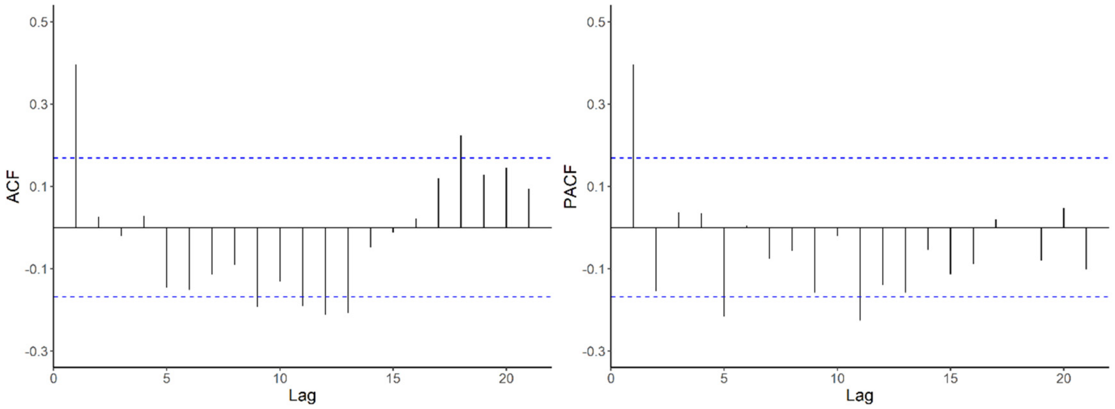

Figure S1. Autocorrelation (left) and partial autocorrelation (right) function of the residuals of the non-linear regression model for temperature anomalies adjusted by year and CO_2 emission

Table and functional form of the model for temperature anomalies with year and CO_2 emission as independent variables

Table S2. Results of the linear regression model for temperature anomalies adjusted for year linearly and CO_2 emission with natural cubic splines with 5 degrees of freedom. The 95% CI was computed using the HAC estimator.

Variable	Coefficient	Standard error
Intercept	-22.140948	5.6256498
Year	0.011681	0.0029903
ns(carbon_emissions. df = 5)1	-0.506030	0.2515340
ns(carbon_emissions. df = 5)2	-0.985015	0.2430262
ns(carbon_emissions. df = 5)3	-0.513098	0.3244648
ns(carbon_emissions. df = 5)4	-1.151119	0.4600711
ns(carbon_emissions. df = 5)5	-0.393499	0.3535838

The functional form of the linear regression model for the expected value of temperature anomalies adjusted for CO_2 emission with natural cubic splines with 5 degrees of freedom and year linearly is given by

$$E(temp_{anomaly}) = X\hat{\beta}.$$

where $X\hat{\beta} =$

$$-22.140948 + 0.01168096 * year - 0.50603025 * [1] - 0.98501459 * [2] - 0.51309841 * [3] - 1.1511193 * [4] - 0.3934988 * [5]$$

and the variable CO_2 emission is pretransformed into the natural cubic spline bases using the *ns* function of the R package *splines* yielding the respective values for [1] - [5].

Sensitivity analysis

Data collected in a time sequence manner typically contains autocorrelated errors since one observation point at a specific time tends to be correlated with its adjacent observation point. Serial correlation of current errors terms with past ones violates the elementary regression model assumption of independent and identically distributed (iid) error terms. This model misspecification can cause distortions in the ordinary least-squares regression procedure similar to the consequences introduced by near collinearity such as biased standard errors of the model parameters.

In order to study the uncertainty inferred by the autocorrelated residuals. a non-linear regression model with ARMA errors was additionally fitted to the climate data example using the auto.arima function of the R-package forecast (31). Again. the independent variable CO_2 emission was included in the modelling process using natural cubic splines with 5 degrees of freedom and year linearly. For the selection of the ARMA parameters p and q. the lowest bias corrected Akaike information criterion (AICc) value was used. The *auto.arima* function suggested a regression model with ARMA(1.0.2) errors as the best fit to the observed data despite the strong evidence of non-stationarity and trend of the time series provided by the Augmented Dickey-Fuller test (TDF=-2.24. p=0.47) and the visual time series display in Figure 2. A possible explanation might lie in the structural breaks within the temperature time series causing standard unit root tests to fail as stated by Estrada and Perron (32). The Ljung–Box test was conducted to examine the presence of autocorrelation among the model residuals and gave no evidence for a rejection of the null hypothesis of independence (TLB=0.01. df=1. p=0.93). The time plot of the residuals. the corresponding ACF. and the distribution of the model residuals were also inspected but did not raise suspicions regarding model violations. Since the estimated regression coefficients of the non-linear regression model with ARMA errors have no straightforward interpretation again only the partial effect plots are illustrated in supplementary figure 2.

Despite the extensive overlap of the partial effect curves corresponding to year and CO_2 emissions. the superimposed partial effect plots generated by the standard regression (reg) approach and the regression with ARMA errors (regARMA) model illustrates an increased uncertainty of the estimated effect curve in the regARMA model as can be seen in supplementary figure 2. The regression model with ARMA(1.2) errors indicates a stronger loss of precision regarding the model parameters than the standard regression approach. Despite these deviations in the spectrum of the confidence interval. the estimated partial effects of both models only show slight differences at the extremes of the data ranges.

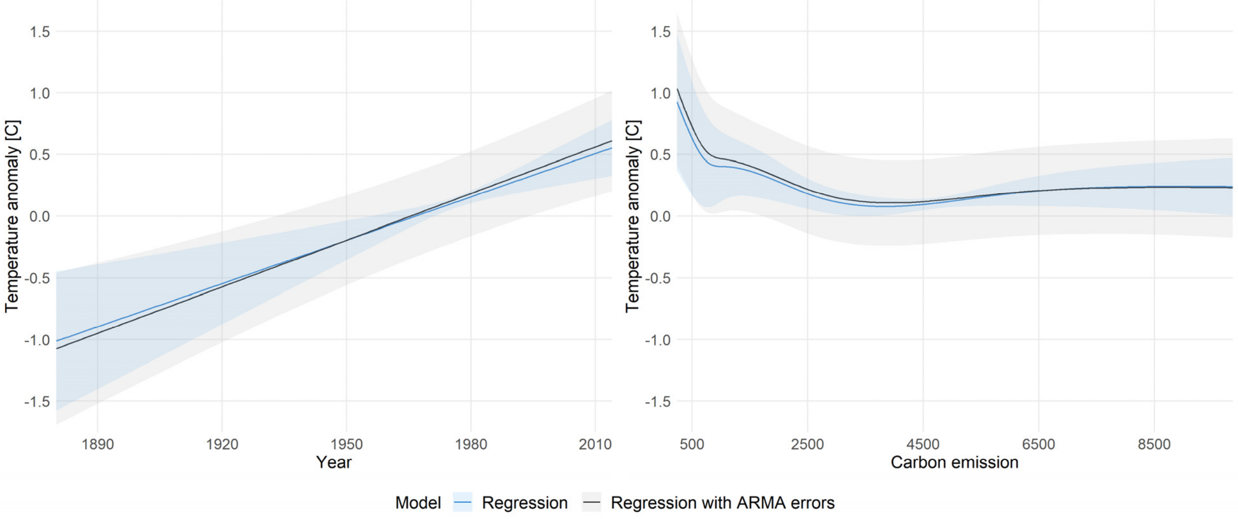

Figure S2. Partial effect plots obtained by classical regression and the regression model using ARMA errors to adjust for the serially correlated error terms.
