# Supplementary material for: Regression with Highly Correlated Predictors: Variable Omission Is Not the Solution
Source: Int J Environ Res Public Health. 2021 Apr 17;18(8):4259. doi: 10.3390/ijerph18084259 (PMC8073086; doi:10.3390/ijerph18084259)
Supplement: Supplementary file 1 [file ijerph-18-04259-s001.zip › Paper___Climate_Change_SuppM2_V10.pdf]

Supplementary Material 2

Regression with highly correlated predictors: variable omission is not the solution

Full R Markdown report on the analyses of the blood analysis example

Read data set

```
tab1<-read.csv2("data_blood.csv")
attach(tab1)
```

Sum of five subtypes equals white blood cell counts

```
sum_cells <- NEU + EOS + BASO + LYM + MONO

plot(sum_cells, WBC)
abline(0,1, lty=3)
```

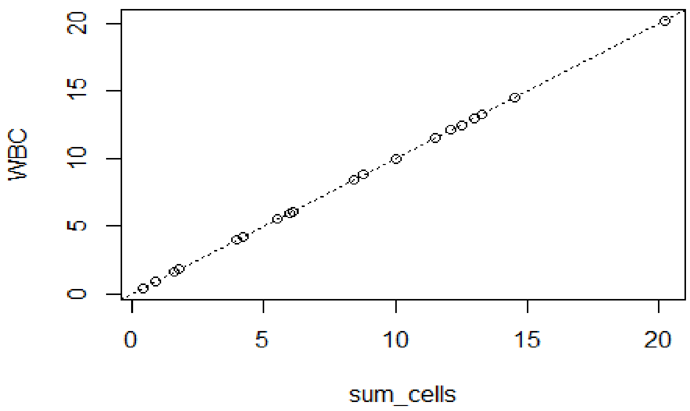

Pairwise correlations

```
round(cor(cbind(NEU, EOS, BASO, LYM, MONO, WBC)),3)

##        NEU   EOS   BASO   LYM  MONO    WBC
## NEU   1.000 0.211 -0.078 0.451 0.732  0.982
## EOS   0.211 1.000  0.170 0.134 0.024  0.218
## BASO -0.078 0.170  1.000 0.053 0.021 -0.045
## LYM   0.451 0.134  0.053 1.000 0.765  0.601
## MONO  0.732 0.024  0.021 0.765 1.000  0.831
## WBC   0.982 0.218 -0.045 0.601 0.831  1.000
```

Exact collinearity

The analysis of the basic regression model will result in the program setting one of the coefficients to NA.

```
mod <- lm(CRP ~ NEU + EOS + BASO + LYM + MONO + WBC)
summary(mod)
```

```
##
## Call:
## lm(formula = CRP ~ NEU + EOS + BASO + LYM + MONO + WBC)
##
## Residuals:
##     Min      1Q  Median      3Q     Max
## -5.8418 -2.3144  0.4375  3.2276  4.5418
##
## Coefficients: (1 not defined because of singularities)
##             Estimate Std. Error t value Pr(>|t|)
## (Intercept)  10.40609    1.82392   5.705 5.43e-05 ***
## NEU           0.65243    0.31278   2.086   0.0558 .
## EOS         -19.47720    7.78284  -2.503   0.0253 *
## BASO         -6.96221   24.28428  -0.287   0.7785
## LYM          -3.08362    2.07602  -1.485   0.1596
## MONO         -0.04194    2.72566  -0.015   0.9879
## WBC                NA         NA      NA       NA
## ---
## Signif. codes:  0 '***' 0.001 '**' 0.01 '*' 0.05 '.' 0.1 ' ' 1
##
## Residual standard error: 3.744 on 14 degrees of freedom
## Multiple R-squared:  0.5387, Adjusted R-squared:  0.3739
## F-statistic: 3.269 on 5 and 14 DF,  p-value: 0.03657
```

Next, VIFs are computed using the package regclass. The function VIF results in an error as there is exact collinearity.

```
try(VIF(mod))
```

```
## Error in VIF(mod) : there are aliased coefficients in the model
```

Near collinearity

Now we consider a model with just two variables, NEU and WBC. First we investigate correlation.

```
plot(NEU, WBC)
abline(0,1,lty=3)
text(0,20, paste("Correlation = ", round(cor(NEU, WBC),3)), adj=0)
legend("bottomrigh", lty=3, legend="Diagonal")
```

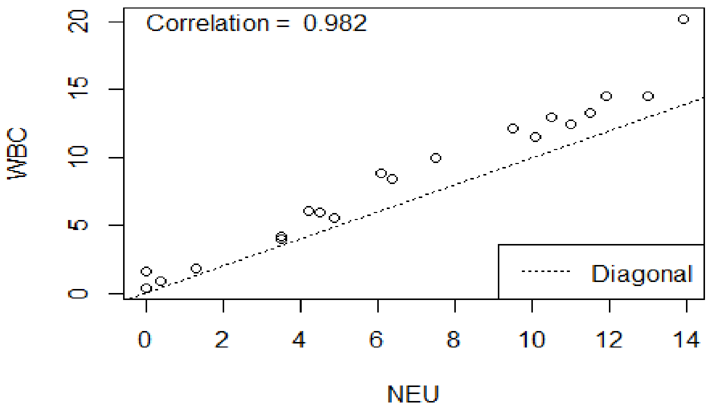

Now we fit the model and evaluate the VIFs.

```
mod2 <- lm(CRP ~ NEU + WBC)
VIF(mod2)

##      NEU      WBC
## 28.72946 28.72946
```

Further, we calculate the condition indices and the variance decomposition proportions

```
 eigprop(mod2)

##
## Call:
## eigprop(mod = mod2)
##
##   Eigenvalues      CI (Intercept)    NEU    WBC
## 1       2.7859  1.0000       0.0308 0.0013 0.0012
## 2       0.2090  3.6514       0.9260 0.0087 0.0064
## 3       0.0051 23.2760       0.0432 0.9900 0.9923
##
## ===============================
## Row 3==> NEU, proportion 0.990009 >= 0.50
## Row 3==> WBC, proportion 0.992348 >= 0.50
```

and perform redundancy analysis

```
redun(~ NEU + WBC)

##
## Redundancy Analysis
##
## redun(formula = ~NEU + WBC)
##
## n: 20    p: 2    nk: 3
```

```
##
## Number of NAs:    0
##
## Transformation of target variables forced to be linear
##
## R-squared cutoff: 0.9    Type: ordinary
##
## R^2 with which each variable can be predicted from all other variables:
##
##   NEU   WBC
## 0.969 0.966
##
## Rendundant variables:
##
## NEU
##
## Predicted from variables:
##
## WBC
##
##   Variable Deleted   R^2 R^2 after later deletions
## 1              NEU 0.969
```

Evaluate the model:

```
summary(mod2)
```

```
##
## Call:
## lm(formula = CRP ~ NEU + WBC)
##
## Residuals:
##     Min      1Q  Median      3Q     Max
## -5.7657 -3.7508 -0.5381  3.6528  6.9263
##
## Coefficients:
##             Estimate Std. Error t value Pr(>|t|)
## (Intercept)    8.979      1.800   4.989 0.000112 ***
## NEU            2.650      1.157   2.290 0.035071 *
## WBC           -1.954      0.957  -2.042 0.056982 .
## ---
## Signif. codes:  0 '***' 0.001 '**' 0.01 '*' 0.05 '.' 0.1 ' ' 1
##
## Residual standard error: 4.256 on 17 degrees of freedom
## Multiple R-squared:  0.2762, Adjusted R-squared:  0.191
## F-statistic: 3.243 on 2 and 17 DF,  p-value: 0.0641
```

The regression coefficients are quite extreme, but this results from their meaning:

The regression coefficient of NEU has the interpretation of the expected difference in CRP corresponding to a difference of 1 G/L in NEU but keeping WBC constant. From the plot above we see that it is sometimes not possible to increase NEU by 1 G/L and at the same time holding WBC fixed, as NEU is a component of WBC and cannot exceed WBC. Hence the regression coefficient has a problematic interpretation.

Remedy: compute a new variable WBCnoNEU = WBC - NEU

```
WBCnoNEU <- WBC - NEU
```

Evaluate correlation again:

```
plot(NEU, WBCnoNEU)
text(0,6, paste("Correlation = ", round(cor(NEU, WBCnoNEU),3)), adj=0)
```

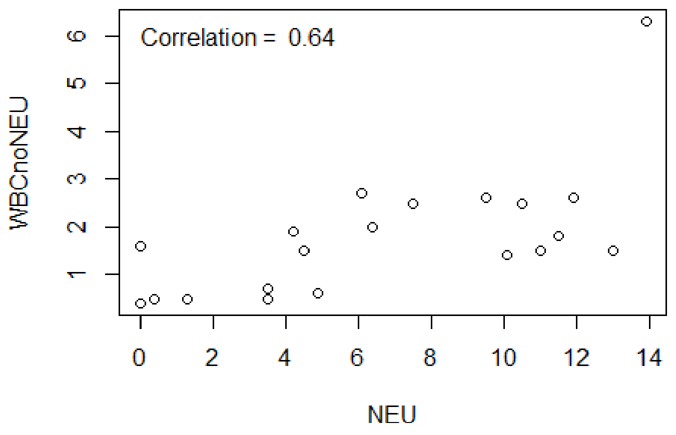

Fit model and evaluate VIFs:

```
mod3 <- lm(CRP ~ NEU + WBCnoNEU)
VIF(mod3)

##      NEU WBCnoNEU
## 1.694783 1.694783
```

Again, calculate the condition indices and the variance decomposition proportions. The condition number (largest condition index) is smaller than 10.

```
eigprop(mod3)

##
## Call:
## eigprop(mod = mod3)
##
##   Eigenvalues     CI (Intercept)    NEU WBCnoNEU
## 1      2.6845 1.0000      0.0336 0.0228   0.0255
## 2      0.2008 3.6559      0.9116 0.0642   0.2773
## 3      0.1147 4.8381      0.0548 0.9130   0.6972
```

```
##
## ================================
## Row 3==> NEU, proportion 0.913003 >= 0.50
## Row 3==> WBCnoNEU, proportion 0.697180 >= 0.50
```

Redundancy analysis states no redundant variables:

```
redun( ~ NEU + WBCnoNEU)

##
## Redundancy Analysis
##
## redun(formula = ~NEU + WBCnoNEU)
##
## n: 20    p: 2    nk: 3
##
## Number of NAs:    0
##
## Transformation of target variables forced to be linear
##
## R-squared cutoff: 0.9    Type: ordinary
##
## R^2 with which each variable can be predicted from all other variables:
##
##      NEU WBCnoNEU
##    0.508    0.419
##
## No redundant variables
```

Evaluate the model:

```
summary(mod3)

##
## Call:
## lm(formula = CRP ~ NEU + WBCnoNEU)
##
## Residuals:
##     Min      1Q  Median      3Q     Max
## -5.7657 -3.7508 -0.5381  3.6528  6.9263
##
## Coefficients:
##             Estimate Std. Error t value Pr(>|t|)
## (Intercept)   8.9788     1.7998   4.989 0.000112 ***
## NEU           0.6961     0.2811   2.477 0.024071 *
## WBCnoNEU     -1.9542     0.9570  -2.042 0.056982 .
## ---
## Signif. codes:  0 '***' 0.001 '**' 0.01 '*' 0.05 '.' 0.1 ' ' 1
##
## Residual standard error: 4.256 on 17 degrees of freedom
```

```
## Multiple R-squared:  0.2762, Adjusted R-squared:  0.191
## F-statistic: 3.243 on 2 and 17 DF,  p-value: 0.0641
```

The standard errors for the coefficient of NEU is now considerably smaller, and the value of the coefficient is quite different from the model above. Still, the multiple R^2 is exactly equal to model mod2. The reason is that the coefficient of NEU now has a different meaning (expected difference in CRP corresponding to a difference in NEU of 1 G/L given constant concentrations of all other components of WBC). However, the coefficient of WBCnoNEU has the same meaning as in Model mod2 (expected difference in CRP corresponding to a difference in WBC of 1 G/L given constant NEU), and hence its value and standard error in model mod3 do not differ from those in mod2.
